# Retraction of: MicroRNA-346 facilitates cell growth and metastasis, and suppresses cell apoptosis in human non-small cell lung cancer by regulation of XPC/ERK/Snail/E-cadherin pathway

**DOI:** 10.18632/aging.205987

**Published:** 2024-08-30

**Authors:** Cheng-Cao Sun, Shu-Jun Li, Zhan-Peng Yuan, De-Jia Li

**Affiliations:** 1 Department of Occupational and Environmental Health, School of Public Health, Wuhan University, Wuhan 430071, P. R. China; 2 Wuhan Hospital for the Prevention and Treatment of Occupational Diseases, Wuhan 430071, P. R. China; 3 Department of Toxicology, School of Public Health, Wuhan University, Wuhan 430071, P. R. China

**Keywords:** microRNA-346 (miR-346), XPC, non-small cell lung cancer (NSCLC), oncogenesis

**This article has been retracted:** Aging has completed its investigation of this paper. We found multiple external duplications of the panels showing colony formation assays in **Figure 5C**. The authors reused previously published data from colony formation assay published in a different study [1] and again used some of the images in a later published article [2]. In addition, there are duplications of plate images from papers from unrelated labs [3, 4]. The corresponding author asked to withdraw/retract this paper and mentioned that: “the first author mislabeled the samples and I lost the confidence in the reliability of the conclusion.” No responses were received from the other authors. Because the validity of the reported results and conclusions is under the question, the Aging Editors retracted this article.
